# Anion-Conducting Polymer Electrolyte without Ether Linkages and with Ionic Groups Grafted on Long Side Chains: Poly(Alkylene Biphenyl Butyltrimethyl Ammonium) (ABBA)

**DOI:** 10.3390/membranes12030337

**Published:** 2022-03-18

**Authors:** Riccardo Narducci, Raul Andres Becerra-Arciniegas, Luca Pasquini, Gianfranco Ercolani, Philippe Knauth, Maria Luisa Di Vona

**Affiliations:** 1 Department of Industrial Engineering and International Laboratory “Ionomer Materials for Energy”, University of Rome Tor Vergata, Via del Politecnico 1, 00133 Roma, Italy; raul-andres.becerra-arciniegas@etu.univ-amu.fr (R.A.B.-A.); divona@uniroma2.it (M.L.D.V.); 2 CNRS, MADIREL (UMR 7246) and International Laboratory “Ionomer Materials for Energy”, Aix-Marseille University, Campus St Jérôme, 13013 Marseille, France; luca.pasquini@univ-amu.fr (L.P.); philippe.knauth@univ-amu.fr (P.K.); 3 Department of Chemical Sciences and Technologies, Via della Ricerca Scientifica, University of Rome Tor Vergata, 00133 Roma, Italy; ercolani@uniroma2.it

**Keywords:** anion exchange membrane, polycondensation, blend membrane, ionic conductivity

## Abstract

In this work we report the synthesis of the new ionomer poly(alkylene biphenyl butyltrimethyl ammonium) (ABBA) with a backbone devoid of alkaline-labile C-O-C bonds and with quaternary ammonium groups grafted on long side chains. The ionomer was achieved by metalation reaction with *n*-butyllithium of 2-bromobiphenyl, followed by the introduction of the long chain with 1,4-dibromobutane. The reaction steps were followed by ^1^H-NMR spectroscopy showing the characteristic signals of the Br-butyl chain and indicating the complete functionalization of the biphenyl moiety. The precursor was polycondensed with 1,1,1-trifluoroacetone and then quaternized using trimethylamine (TMA). After the acid catalyzed polycondensation, the stoichiometric ratio between the precursors was respected. The quaternization with TMA gave a final degree of amination of 0.83 in agreement with the thermogravimetric analysis and with the ion exchange capacity of 2.5 meq/g determined by acid–base titration. The new ionomer blended with poly(vinylalcohol) (PVA) or poly(vinylidene difluoride) (PVDF) was also characterized by water uptake (WU) and ionic conductivity measurements. The higher water uptake and ionic conductivity observed with the PVDF blend might be related to a better nanophase separation.

## 1. Introduction

The synthesis of strong, durable, and high ion conducting anion exchange membranes (AEM) is a promising subject of study for various applications such as fuel cells (FC), water and CO_2_ electrolyzers, and other energy storage and conversion devices [1,2,3,4]. Anion exchange membrane fuel cells (AEMFC) present several advantages in comparison with proton exchange membrane fuel cells: (i) the faster kinetics of the oxygen reduction reaction (ORR) allows a reduced amount or the elimination of noble metal electrocatalysts, (ii) small organic molecules such as methanol and ethanol can be used as fuels, (iii) less corrosion problems [5,6]. Furthermore, AEM are less expensive than common perfluorinated ionomers, such as long side chain (LSC) Nafion or short side chain (SSC) Aquivion [7,8].

Unfortunately, the inferior ionic conductivity due to the lower mobility of OH^−^, the minor phase separation, the low stability at high pH and temperatures above 80 °C, and the risk of carbonation due to the presence of CO_2_ in the air have been major barriers to the use AEMFC as reliable clean energy technology [9,10,11]. In recent years, a great variety of polymer backbones were proposed [12,13] including poly(arylene ether sulfones) [14], poly(phenylene oxide) (PPO) [15,16], and poly(benzimidazole) [17]. AEM were usually prepared by post-functionalization, for example via chloromethylation followed by quaternization with tertiary amines such as trimethylamine (TMA) and 1,4-diazabicyclo[2.2.2]octane (DABCO). However, the random functionalization requires an optimization of the desired degree of amination and the process scalability for industrial use is not very advantageous [10,18]. 

One of the main roles of the polymer backbone in AEM is to guarantee the mechanical stability by the interaction between chains by Van der Waals forces, by entanglements or by crystallinity [19,20,21,22]. The low chemical stability in alkaline media is a drawback: for example, in the case of polysulfone (PSU) the degradation in specific positions is promoted by the inductive effect of the sulfone groups [23]. AEM based on PPO are expected to have a higher stability because they present a less activated backbone [24], but the presence of ether linkages, although improving the solubility in common organic solvents, makes AEM based on PPO weak in high pH environments, because the attack by the strong OH^−^ nucleophile on the ether groups causes the degradation of the polymers [25]. The alkaline attack may occur in several ways [9,11,24]: the most difficult to avoid is the S_N_2 reaction, whose rate is proportional to the residual ion exchange capacity (IEC) and the OH^−^ concentration [23,26]. The E2 elimination produces an alkene when a hydrogen is available in β position [27]. Different strategies are proposed to mitigate the damaging effect of alkaline media [2,15,28,29,30,31,32], including a delocalization of the positive charge [10,29], a protection by steric hindrance [10,33], an introduction of a long chain to separate the charge from the backbone [28,34,35], a different architecture of the polymer backbone [31,36,37,38], or the formation of composites with inorganic materials stable in alkaline environments [39,40]. Theoretical studies by Pivovar and co-workers show that the most stable side chain length corresponds to four to five CH_2_ groups [41]; most recently the comparison between benzyltrimethylammonium and long alkyl-tethered quaternary ammonium (hexyltrimethylammonium) shows a better chemical stability of the latter in alkaline conditions [42]. Other experimental and modeling studies of AEM based on PPO with trimethylpentylammonium side groups show that the stability in alkaline solution is clearly improved versus short side chain ionomers [43]. 

Bae and coworkers propose a new one-pot, metal-free, tunable synthetic route of aromatic polymers by acid catalyzed polycondensation of biphenyl and trifluoromethyl alkyl ketones [44,45,46]. The use of acidic media allows the polycondensation of the monomers containing a bromoalkyl side chain without affecting the quaternary ammonium precursor [47]. The main feature is the absence of oxygen in the backbone, giving it greater stability in a basic environment without compromising the ductility. The related ionomer presents a well-controlled degree of functionalization; in addition, the reaction was conducted in absence of toxic reagents. These polymers are insoluble in water, flexible, chemically and mechanically stable, and show excellent long-term alkaline stability at 80 °C [44]. The acid catalyzed polycondensation is reported to afford high molecular weight [44,48]. 

In this work, the aim is to use the concept of a backbone with only C-C bonds and without ether groups by combining it with the introduction of a long aliphatic chain linked to the aromatic part with a grafted ammonium group at the end of the alkyl spacer. Furthermore, planar, π-conjugated phenyl groups can inhibit the electrode activity by adsorption; the functionalization of the phenyl groups with a bulky side chain having a certain steric hindrance can also limit this adverse adsorption effect on electrocatalytic reactions [49]. Moreover, the presence of a substituent on the phenyl ring can inhibit the formation of phenols, which limits the ORR activity [49]. 

The first step is the metalation reaction with butyllithium of 2-bromobiphenyl, followed by the introduction of a long chain with 1,4-dibromobutane. This precursor is poly-condensed with 1,1,1-trifluoroacetone and then quaternized using TMA. The resulting new ionomer poly(alkylene biphenyl butyltrimethyl ammonium) (ABBA) is blended with poly(vinylalcohol) (PVA) [40,50] or poly(vinylidene difluoride) (PVDF) [51,52]. PVA is chosen because of its nontoxicity, film forming properties and low cost, and PVDF for its low reactivity, high solubility in polar solvents and the ability to enhance the nanophase separation [53,54,55]. The samples are characterized by NMR spectroscopy, water uptake (WU), ion exchange capacity (IEC), and ionic conductivity measurements. The ability to modulate the polycondensation reaction of the new ionomer ABBA combined with the good solubility in common organic solvents, the high water uptake, and the compatibility with other polymers (blend membranes) makes it a serious candidate for both AEM and ionomer electrode materials.

## 2. Materials and Methods

### 2.1. Materials

2-Bromobiphenyl and 1,4-dibromobutane were reagent grade and purchased from Fluorochem (Hadfield, UK). *n*-Butyllithium (BuLi, 2.5 M in hexane), tetrahydrofuran (THF, anhydrous), dichloromethane (DCM), 1,1,1-trifluoroacetone, trifluoromethanesulfonic acid (TFSA), trimethylamine (TMA, 4.2 M in ethanol), poly(vinyl alcohol) (PVA, Mw = 89–98,000, 99% hydrolyzed), polyvinylidene difluoride (PVDF, Mw = 534,000), glutaraldehyde (GA) (50 wt% in water), *N*-methyl-2-pyrrolidone (NMP), and methanol (anhydrous) were reagent grade and purchased from Sigma-Aldrich (Milano, Italy). All the reagents were used as received.

### 2.2. Synthesis

#### 2.2.1. Synthesis of 2-(4-bromobutyl)biphenyl)(2)

An amount of 1.4 g of 2-bromobiphenyl (**1**, 6.0 mmol) was added under nitrogen flux to 5 mL of anhydrous THF. The resulting solution was cooled to −78 °C and BuLi was introduced (7.8 mmol). After 2 h at room temperature (RT), the solution was cooled again to −78 °C and then 1,4-dibromobutane (15.6 mmol) was added. The reaction mixture was left at ambient temperature for 12 h, dried 3 h under vacuum and heated in an oven at 70 °C for 48 h. Yield 27%. ^1^H NMR (CDCl_3_): 1.6 (BiPh-CH_2_CH_2_CH_2_CH_2_Br, m, 2H), 1.8 (BiPh-CH_2_CH_2_CH_2_CH_2_Br, m, 2H), 2.6 (BiPh-CH_2_CH_2_CH_2_CH_2_Br, t, 2H), 3.3 (BiPh-CH_2_CH_2_CH_2_CH_2_Br, t, 2H), 7.2–7.5 (aromatic region, m, 9H).

#### 2.2.2. Synthesis of the Precursor poly[(2-(4-bromobutyl)biphenyl-4,4′-diyltrifluoromethylmethylmethylene] (3)

An amount of 0.6 g of **2** (2.1 mmol), 0.19 g 1,1,1-trifluoroacetone (2.1 mmol), 2 mL DCM and 2 mL TFSA (22 mmol) were stirred at room temperature under nitrogen flux [56]. The resulting dark brown solution was left 12 h at RT, then poured slowly into methanol and white-grey fibers precipitated. The polymer **3** was filtered, washed with hot methanol, dried in a vacuum pump and maintained under P_2_O_5_. Yield 51%. ^1^H NMR (CDCl_3_): 1.6–1.8 (-BiPh-CH_2_CH_2_CH_2_CH_2_Br, 4H), 2.0 (-(CF_3_)(CH_3_)C-BiPh, 3H), 2.6 (-BiPh-CH_2_CH_2_CH_2_CH_2_Br, 2H), 3.3 (-BiPh-CH_2_CH_2_CH_2_CH_2_Br, 2H), δ = 7.2–7.7 (aromatic region, m, 9H). 

#### 2.2.3. Synthesis of the Target Polymer ABBA, namely poly[2-(*N,N,N*-trimetylbutane-1-aminium-4-yl)biphenyl-4,4′-diyltrifluoromethyl methylmethylene] (**4**)

An amount of 0.5 g of **3** (1.5 mmol) was dissolved in NMP and TMA was added in excess (3.6 mmol) because of the well-known TMA volatility. The solution was kept at 70 °C for 72 h and dried at the vacuum pump for 3 h to remove the excess of TMA. Yield 83%. ^1^H NMR (DMSO-d_6_): 1.4–1.5 (-BiPh-CH_2_CH_2_CH_2_CH_2_-N^+^, 4H), 1.9 (-(CF_3_)(CH_3_)C-BiPh-, 3H), 2.6 (-BiPh-CH_2_CH_2_CH_2_CH_2_-N^+^, 2H), 2.9 (-BiPh-CH_2_CH_2_CH_2_CH_2_-N^+^(CH_3_)_3_, s, 9H), 3.1 (-BiPh-CH_2_CH_2_CH_2_CH_2_-N^+^, 2H), 7.0–7.5 (aromatic region, m, 9H). 

#### 2.2.4. Blend Membranes

##### PVA

An amount of 20 mg of PVA (20% by weight with respect to ABBA) was dissolved under strong stirring (600 rpm) in bidistilled water at 80 °C for 10 min. A hot ABBA solution (80 mg in 5 mL of NMP) was suddenly added to the PVA solution and mixed for about 10 min. Then, glutaraldehyde (5% by weight with respect to the PVA) was added. Later, 4 drops of concentrated HCl were inserted and the stirring was maintained for 10 min at RT.

##### PVDF

An amount of 30 mg of PVDF (30% by weight with respect to ABBA) was dissolved in 5 mL of NMP under stirring. A hot ABBA solution (70 mg in 4 mL of NMP) was suddenly added to the PVDF solution and mixed for about 10 min.

##### Blend Membrane Casting 

The solutions were poured into a Teflon Petri dish and then placed in an oven at 85 °C for 3 days. The membranes were peeled off, washed with bidistilled water and then exchanged with 2 M KCl for 2 days at RT to obtain the chloride form. The membranes were washed again in bidistilled water to eliminate the excess of KCl and stored over P_2_O_5_. The used percentages of PVA and PVDF gave the best membranes from a handling point of view. We realized various membranes with a diameter from 2 to 5 cm and thickness from 50 to 150 μm. 

### 2.3. Characterization Techniques

#### 2.3.1. ^1^H-NMR Spectroscopy

^1^H-NMR spectra were recorded with a Bruker Avance 400 spectrometer (Milano, Italy) operating at 400.13 MHz using deuterating solvents (CDCl_3_, DMSO-d_6_).

#### 2.3.2. Thermogravimetric Analysis 

The high-resolution thermogravimetric analysis was performed with a TA Q500 apparatus (TA instruments, New Castle, DE, USA). The experiments were executed between 30–700 °C in air with a maximum heating rate of 3 K/min.

#### 2.3.3. Ion Exchange Capacity 

The IEC (milliequivalents per gram of dry polymer) was determined by potentiometric acid–base titration. The membranes were washed in bidistilled water at 60 °C for 2 days to remove residual salts. After drying over P_2_O_5_ for 72 h, the membranes were weighed and immersed in a 0.02 M HCl solution. The acid solution was then back-titrated with 0.02 M NaOH solution.

#### 2.3.4. Water Uptake 

The water uptake (%) was determined at 25 °C following the equation:

(1) $$WU=100\cdot \frac{m_{wet}-m_{dry}}{m_{dry}}$$

*m_dry_* is the mass of dried sample and *m_wet_* is the mass of wet sample after immersion in bidistilled water for 48 h. Before weighing, the excess of water on the sample surface was carefully removed with absorbing paper.

#### 2.3.5. Ionic Conductivity

The samples in Cl^−^ form were obtained by immersion in 2 M NaCl solution for 48 h and washed carefully with bidistilled water. They were analyzed in fully hydrated conditions by impedance spectroscopy (VSP-300, Bio-Logic Science Instruments, Seyssinet-Pariset, France) between 6 MHz and 1 Hz with a signal amplitude of 20 mV inside a Swagelok cell with stainless steel electrodes. The resistance *R* of the membranes was obtained from Nyquist plots at 25 °C, 45 °C, 60 °C and 80 °C, using non-linear least square fitting with an equivalent circuit of a resistance *R* in series with a parallel circuit resistance/constant phase element. The ionic conductivity was determined following the equation:

(2) $$\sigma =\frac{d}{R\times A}$$

*d* is the thickness of the membranes (80 μm) and *A* is the electrode area (0.264 cm^2^).

## 3. Results and Discussion

**Figure 1:**
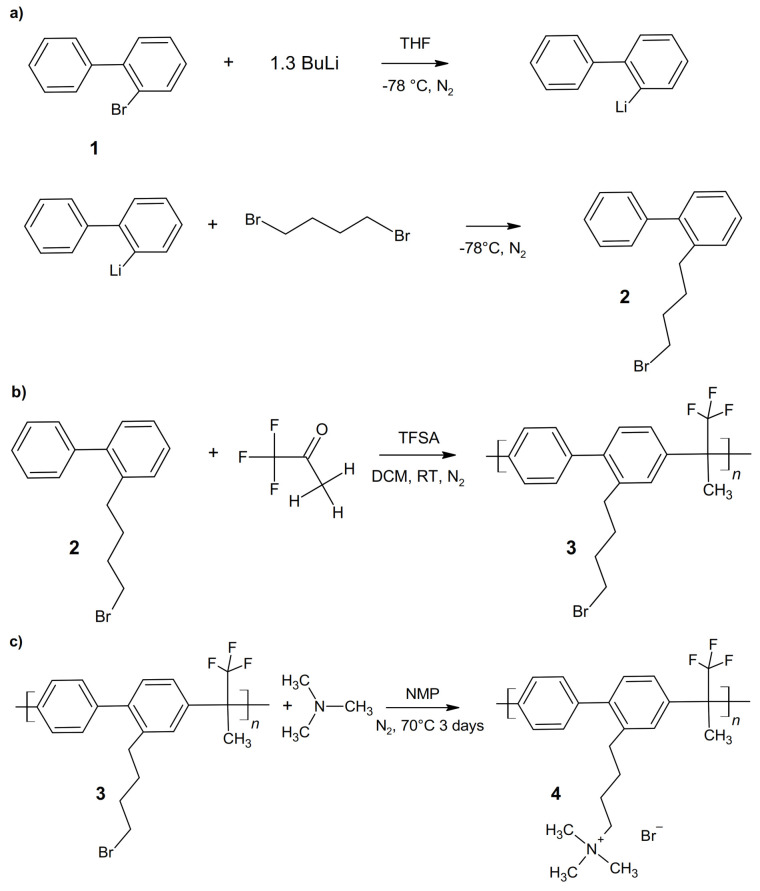
Synthesis scheme: (**a**) 2-(4-bromobutyl)biphenyl (**2**); (**b**) poly[(2-(4-bromobutyl)biphenyl-4,4′-diyltrifluoromethylmethylmethylene] (**3**); (**c**) ABBA (**4**).

The synthesis scheme is shown in Figure 1. The first step of the synthesis is the formation of the long side chain precursor via metalation reaction with butyllithium of 2-bromobiphenyl, followed by the introduction of the long chain with 1,4-dibromobutane. The two reactions are carried out at low temperature (−78 °C) and under nitrogen flux (Figure 1a) [57]. 

The aromatic main chain polymer is prepared by acid catalyzed Friedel–Crafts polycondensation of **2** and 1,1,1-trifluoroacetone in a stoichiometric ratio of 1:1 at room temperature and under nitrogen flux. The possibility of modulating the ratio makes the reaction very versatile (Figure 1b). 

The bromobutyl group on the side chain of the intermediate polymer **3** is converted to *N,N,N-*trimetylbutane-1-aminium-4-yl bromide via S_N_2 reaction with TMA (ABBA) (Figure 1c).

The reaction yields are for the introduction of the side chain (two-step reaction a) at 27% and for the polycondensation (reaction b) at 51%. The final degree of amination (DAM) is 83%.

**Figure 2:**
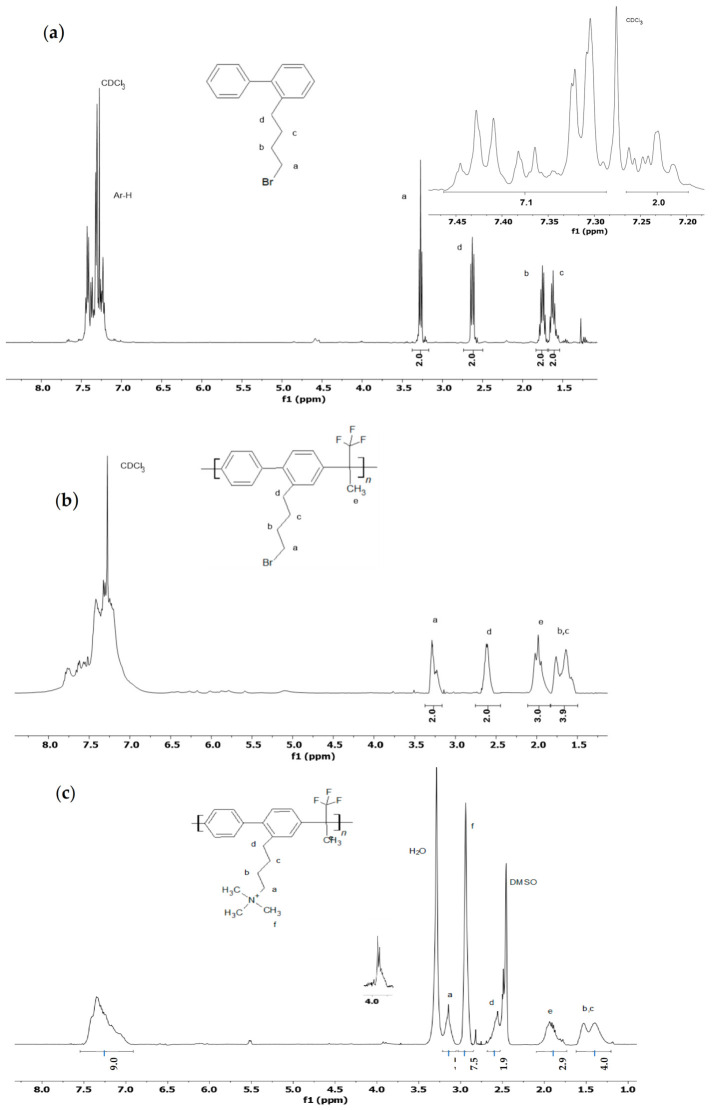
^1^H-NMR analysis of: (**a**) 2-(4-bromobutyl)biphenyl (**2**) in CDCl_3_; (**b**) poly[(2-(4-bromobutyl)biphenyl-4,4′-diyltrifluoromethylmethylmethylene] (**3**) in CDCl_3_; (**c**) PBBAA (**4**) in DMSO-d_6_.

Figure 2 reports the ^1^H-NMR analysis of the synthesis. The spectrum of the functionalized biphenyl (**2**, Figure 2a) shows the characteristic signals of the Br-butyl chain grafted to the aromatic ring. More specifically, the peak at 3.3 ppm due to the terminal -CH_2_Br group (*a*), the benzylic hydrogens at 2.6 ppm (*d*), and the internal peaks of the chain at 1.8 (*b*) and 1.6 (*c*) ppm. The aromatic hydrogens (7.2–7.5 ppm) overlap with the signal of the solvent, making their integration difficult. However, as shown in the insert of Figure 2a excluding the area of CDCl_3_, it is possible to measure the ratio between the protons of the chain and the aromatic protons, indicating a complete functionalization of the biphenyl moiety.

The spectrum of the intermediate, obtained after the acid catalyzed polycondensation, is reported in Figure 2b. The polymer **3** shows the peaks of the long chain (*a–d*) in the same position; the larger base line indicates the formation of a polymer. The new peak at 2.0 ppm (*e*) is ascribed to the CH_3_ group bonded to the quaternary carbon, related to 1,1,1-trifluoroacetone, showing that the reaction has taken place. The observed multiplicity may be due to the long-range coupling between fluorine and the methyl group [58]. By assigning an area of 2H for protons d, the CH_3_ (*e*) area is in perfect agreement with a stoichiometric ratio between the precursors of 1 to 1. The ratio between the hydrogens in α to Br (*d*) and in α to the aromatic ring (*a*) excludes the formation of fused hydrocarbons by intramolecular Friedel–Crafts reaction that was reported in Ref. [57]. 

The spectrum in Figure 2c shows the product of the amination step: ABBA. The signals *b* (1.5 ppm) and *c* (1.4 ppm) of the butyl moiety and the signal *e* (1.9 ppm) of the methyl group in the main chain were shifted with respect to the previous spectra due to the presence of the ammonium group and the different deuterated solvent. The signal *d* (2.6 ppm) remains unchanged, while a new peak corresponding to the CH_2_ in α to the ammonium group is centered at 3.1 ppm. The corresponding CH_3_ signal (*f*) is at 2.9 ppm. The degree of amination (DAM) can be evaluated by assigning a value of 9H to the aromatic portion, and the area of the methyl groups linked to the ammonium atom assumes a value of 7.5H corresponding to a DAM of 0.83. In the spectrum a signal can also be recognized due to the CH_2_OH portion (3.9 ppm, insert of Figure 2c), probably due to the hydrolysis of the remaining alkylbromine. 

**Figure 3:**
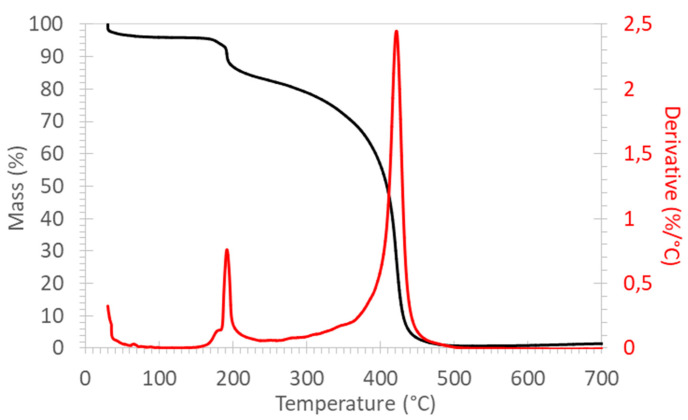
Thermogravimetric analysis of ABBA in air.

The thermogravimetric curve of ABBA is shown in Figure 3. One can recognize three main mass losses. The first one below 100 °C is related to the evaporation of water from the hydrophilic ionomer. The second mass loss of about 12% with a peak maximum at 190 °C corresponds to the removal of the trimethylammonium groups as previously reported for PSU-based ionomers with long side chains [43]; the percentage is in agreement with the degree of amination (DAM = 0.83) of the ionomer. The third mass loss with a peak maximum at 420 °C is attributable to the degradation of the highly thermostable ionomer backbone. This section may be divided by subheadings. It should provide a concise and precise description of the experimental results, their interpretation, as well as the experimental conclusions that can be drawn.

The IEC of the ionomer determined by acid–base titration (IEC = 2.5 meq/g) is in good agreement with the degree of functionalization (DAM) obtained from NMR spectroscopy (IEC 2.4 meq/g), according to the equation:

(3) $$\mathrm{EC}=\frac{\mathrm{DAM}}{M\left(3\;\mathrm{without}\;\mathrm{Br}\right)+\mathrm{DAM}*M\left(\mathrm{TMA}\right)}$$

The water uptakes of blend membranes with 20 wt% PVA or 30 wt% PVDF are 74% and 141%, respectively. The water uptake is lower with hydrophilic PVA than with more hydrophobic PVDF, certainly due to the partial cross-linking of the PVA blend with glutaraldehyde. In order to verify the absence of artefacts, due, e.g., to the loss of residual solvent in the membranes, the anhydrous mass was measured before and after the water uptake and the results were coherent with a difference of less than 7% between the two values. 

The ion conductivity of the two blend membranes is reported between 25 and 80 °C in Table 1; the chloride form is investigated to totally exclude the influence of carbon dioxide. Consistently, the blend membrane with the higher water uptake also presents a higher ionic conductivity, because the ion mobility is larger in highly hydrated conditions, as previously demonstrated for various AEM [59]. The ion conductivity remains modest although the IEC is quite high, indicating a low anion mobility possibly due to an insufficient connectivity of ion-conduction paths in the composite membranes. A possible strategy to overcome this limitation is to improve the phase separation, perhaps by adding longer spacers or changing the TMA with other groups, such as piperidine [60,61]. Furthermore, one could explore blends with more flexible polymers or better organized composites.

**Figure 4:**
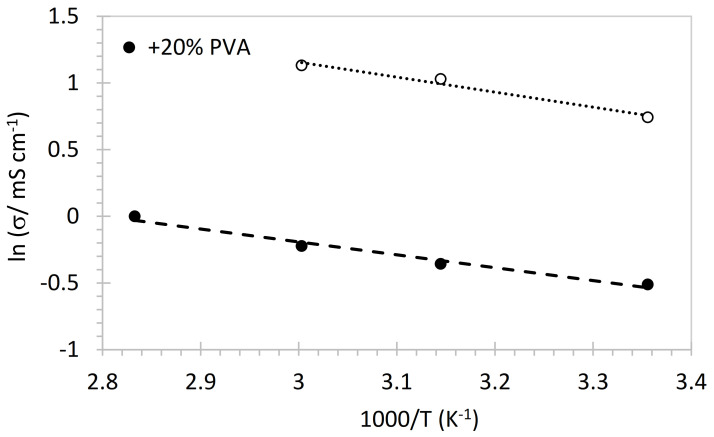
Arrhenius representation of conductivity data of blend membranes.

The activation energy c*E_A_* can be determined from the temperature dependence of the ionic conductivity *σ* (Figure 4) according to the Arrhenius equation:

(4) $$\ln\sigma =A-\frac{E_{A}}{RT}$$

The activation energy (0.08 eV with PVA and 0.10 eV with PVDF) is compatible with ion conduction in aqueous solution, indicating that PVA and PVDF participate in the ionic transport. Similar activation energies were reported for PVA-based gel polymer electrolytes with KOH [62] and quaternized PVA [50]. The activation energies for PVDF-based AEM in the literature are generally somewhat higher, indicating here a particularly favored pathway for ion conduction that might be related to an improved nanophase separation by the presence of hydrophobic PVDF [52]. 

## 4. Conclusions

A new ionomer poly(alkylene biphenyl butyltrimethyl ammonium) (ABBA) without ether linkage in the backbone and with quaternary ammonium groups tethered on long alkyl side chains was prepared by acid catalyzed polycondensation. The reaction is efficient and well controllable. The introduction of a side chain between the aromatic moiety and the positive charge attenuates the polarization effects of the main chain while preserving the matrix. The ability to modulate the backbone and the ionic groups in a stoichiometric way allows reaching high IEC values such as 2.5 meq/g for ABBA. The solubility in organic solvents such as NMP and the compatibility with different polymers such as PVA and PVDF in significant quantities of 20 and 30%, respectively, made homogeneous membranes available. The ability to form stable solutions over several months, combined with the spacing of the positive charge from the backbone, the flexibility due to the quaternary carbon in the matrix and the high IEC, allows us to consider this ionomer a good candidate as an electrode binder and AEM.

## Figures and Tables

**Table 1:** Ionic conductivity (Cl^−^) of blend membranes with PVA and PVDF.

Temperature (°C)	Conductivity (mS cm^−1^)	
	+20% PVA	+30% PVDF
25	0.6	2.1
45	0.7	2.8
60	0.8	3.1
80	1.0	-
